# Novel mutations in hyper‐IgM syndrome type 2 and X‐linked agammaglobulinemia detected in three patients with primary immunodeficiency disease

**DOI:** 10.1002/mgg3.1552

**Published:** 2020-12-30

**Authors:** Xihui Chen, Fangfang Liu, Lijuan Yuan, Meng Zhang, Kun Chen, Yuanming Wu

**Affiliations:** ^1^ Department of Biochemistry and Molecular Biology The Fourth Military Medical University Xi’an China; ^2^ Institute of Neurosciences The Fourth Military Medical University Xi'an, Shaanxi China; ^3^ Department of General Surgery Tangdu Hospital The Fourth Military Medical University Xi’an China; ^4^ Department of Anatomy, Histology and Embryology and K.K. Leung Brain Research Centre The Fourth Military Medical University Xi'an China

**Keywords:** hyper‐IgM syndrome type 2, molecular diagnosis, next‐generation sequencing, primary immunodeficiency diseases, X‐linked agammaglobulinemia

## Abstract

**Background:**

Ambiguous or atypical phenotypes can make a definite diagnosis of primary immunodeficiency diseases based on biochemical indices alone challenging. Further, mortality in early life because of infections in patients with these conditions supports the use of genetic tests to facilitate rapid and accurate diagnoses.

**Methods:**

Genetic and clinical analyses of three unrelated Chinese children with clinical manifestations of recurrent infections, who were considered to have primary immunodeficiency diseases, were conducted. Patient clinical features and serum immunological indices were recorded. Next‐generation sequencing was used to screen for suspected pathogenic variants. Family co‐segregation and in silico analysis were conducted to evaluate the pathogenicity of identified variants, following the American College of Medical Genetics and Genomics guidance.

**Results:**

All three patients were found to have predominant antibody defects. Sequencing analysis revealed that one had two compound heterozygous variants, c.255C>A and c.295C>T, in the autosomal gene, activation‐induced cytidine deaminase (*AICDA*). The other two patients were each hemizygous for the variants c.1185G>A and c.82C>T in the Bruton's tyrosine kinase (*BTK*) gene on the X chromosome. In silico analysis revealed that identified substituted amino acids were highly conserved and predicted to cause structural and functional damage to the proteins.

**Conclusion:**

Four pathogenic variants in *AICDA* and *BTK* were confirmed to cause different forms of hyper‐IgM syndrome type 2 (HIGM2) and X‐linked agammaglobulinemia (XLA); two were novel mutations that have never been reported previously. This is the first report of HIGM2 caused by *AICDA* deficiency in a patient from the Chinese mainland.

## Graphical abstract

In this paper, comprehensive clinical characteristics of the PIDDs patients are recorded, which will give more evidence for the diagnosis of the two diseases. Two novel mutations have never been reported before were identified, which will enrich the worldwide spectrum of HIGM2 and XLA. Moreover, this is the first report of HIGM2 caused by AICDA deficiency in the Chinese mainland cohorts, which will set a good example for similar cases in the future.

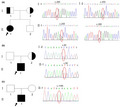

## 1 INTRODUCTION

Primary immunodeficiency diseases (PIDDs) are genetic immune disorders, or congenital immune system dysplasia diseases, leading to an increased susceptibility to infections and autoimmunity (Modell et al., 2018). To date, more than 400 inborn errors of immunity causing PIDDs have been recognized, according to a report from the International Union of Immunological Societies Expert Committee (Tangye et al., 2020). Among them, more than 60% of cases are associated with antibody defects (Al‐Herz et al., 2014).

Hyper‐IgM syndrome type 2 (HIGM2, OMIM 605258) and X‐linked agammaglobulinemia (XLA, OMIM 300755) are both rare PIDDs caused by antibody defects. The prevalence of HIGM2 is 1.5/1,000,000 in Finland and 2/10,000,000 in North America (Minegishi et al., 2000; Trotta et al., 2016), while XLA occurs in 1/100,000 to 1/285,000 and 1/379,000 live births in Norway and the United States, respectively (Stray‐Pedersen et al., 2000; Winkelstein et al., 2006).

In this study, based on a combination of clinical data and genetic analysis, one female and two male patients were diagnosed with HIGM2 and XLA, caused by the deficiency of *AICDA* (NC_000012.12) and *BTK* (NC_000023.11), respectively. Four different causative variants were confirmed using next‐generation sequencing (NGS) technology and bioinformatics analysis, two of which were novel. This is the first report of the diagnosis of patients from the Chinese mainland with HIGM2 by the analysis of variants in *AICDA*.

## 2 PATIENTS AND METHODS

### 2.1 Ethical compliance

This study was approved by an ethics committee.

### 2.2 Patient no. 1

An 8‐year‐old girl was admitted into the hospital because of recurrent cough and expectoration, with no known cause. Chest computed tomography scan revealed inflammation in the upper lobe of the left lung and lesions indicating inflammatory changes in the thickened left lower lobar bronchus. She had been hospitalized four times, also due to lobar pneumonia, within the previous 2 years. Immunological investigations indicated low serum IgA (IgA <26.3 mg/dl; normal range, 85–171 mg/dl) and IgG (IgG, 8.6 mg/dl; normal range, 791–1307 mg/dl), but increased serum IgM (IgM, 910 mg/dl; normal range, 12–226 mg/dl). The patient had developed normally and had no known family history. No abnormal conditions have been observed in her 1‐year‐old brother to date.

### 2.3 Patient no. 2 and no. 3

Patient no. 2 and patient no. 3 were boys of 3 and 6 years old, respectively. They are both sporadic cases without a family history. They began suffering recurrent bacterial infection at 8 months and 1 month after birth, respectively, both without a family history. Low levels of circulating B cells (10% and <0.1%, respectively) and decreased serum IgA, IgG, and IgM levels were detected in both patients. Comparison of the common clinical symptoms shared by these two patients indicated that patient no. 2 presented with relatively mild disease.

### 2.4 Genotyping by NGS and Sanger sequencing

Patient genomic DNA was isolated from peripheral blood samples, using standard methods. Targeted sequence capture was applied by library preparation, solution hybridization, and bead capture using an antibody defect‐associated immunodeficiency screening panel (Table S1). After enrichment of exonic and adjacent intron sequences, NGS (Illumina HiSeq 2000) was performed, and the resulting sequences were aligned against reference sequences from the CCDS (http://www.ncbi.nlm.nih.gov/CCDS/) and dbSNP (http://www.genenames.org) databases to screen for potential disease‐causing variants. Suspected disease‐related variants were then verified by Sanger sequencing and family co‐segregation analyses.

### 2.5 In silico analysis of candidate variants

The effects of amino acid substitutions were predicted using SIFT (http://sift.jcvi.org) and PolyPhen‐2 (http://genetics.bwh.harvard.edu/pph2/), and changes in protein stability related to sequence variants were assessed using I‐Mutant2.0 (http://folding.biofold.org/cgi‐bin/i‐mutant2.0.cgi). Analysis of the conservation of single amino acid sites was conducted using the MEGA software. Changes in polypeptide conformation caused by amino acid substitutions were simulated using SWISS‐MODEL (https://swissmodel.expasy.org).

## 3 RESULTS

### 3.1 Sequence and family co‐segregation analyses

The results of NGS revealed that patient no. 1 had compound heterozygous variants, including missense (c.255C>A, p.Ser85Arg) and nonsense (c.295C>T, p.Arg99ter) alterations in exon 3 of *AICDA* (NM_020661.4). Patient no. 2 had a missense variant (c.82C>T, p.Arg28Cys) in exon 2 and patient no. 3 had a nonsense variant (c.1185G>A, p.Trp395ter) in exon 14 of *BTK* (NM_000061.2).

**FIGURE 1:**
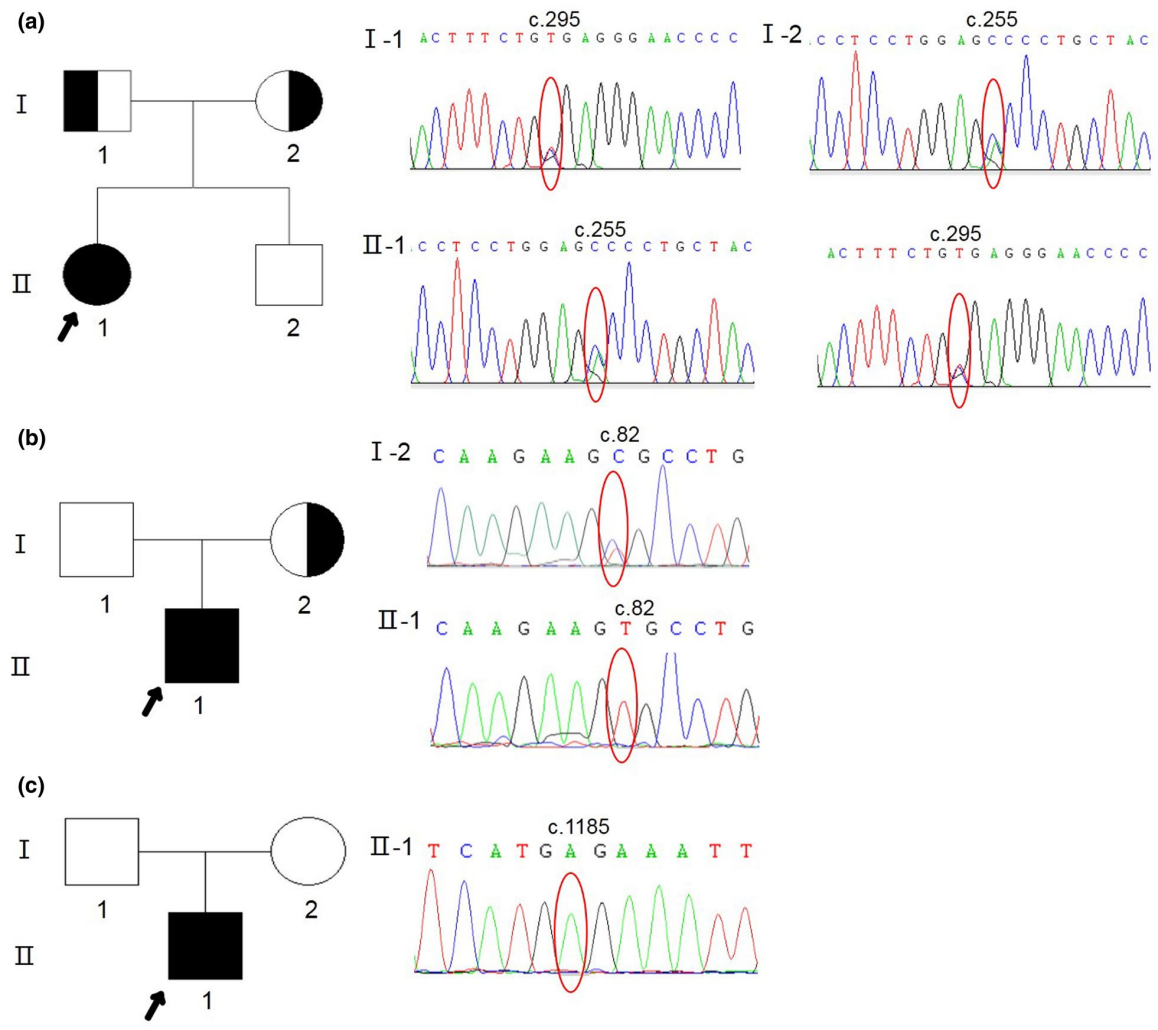
Pedigrees and co‐segregation results for the three families. (a) Patient no. 1 (II‐1) had compound heterozygous mutations, c.255C>A and c.295C>T, in *AICDA*; c.255C>A was inherited from her mother (I‐2) and c.295C>T from her father (I‐1). (b) The c.82C>T mutation in *BTK* in patient no. 2 (II‐1) was inherited from his mother (I‐2). (c) The c.1185G>A mutation of *BTK* in patient no. 3 (II‐1) was not found in either of his parents

All sequence alterations were subsequently confirmed by Sanger sequencing. Further, family co‐segregation analysis demonstrated that the c.255C>A and c.295C>T alleles in patient no. 1 were maternal and paternal, respectively (Figure 1a). The mother of patient no. 2 was heterozygous for the c.82C>T variant, while the nonsense alteration c.1185G>A in patient no. 3 was a de novo change found in neither his father nor his mother (Figure 1b,c).

### 3.2 Structure‐function correlation of *AICDA* and *BTK* variants

**FIGURE 2:**
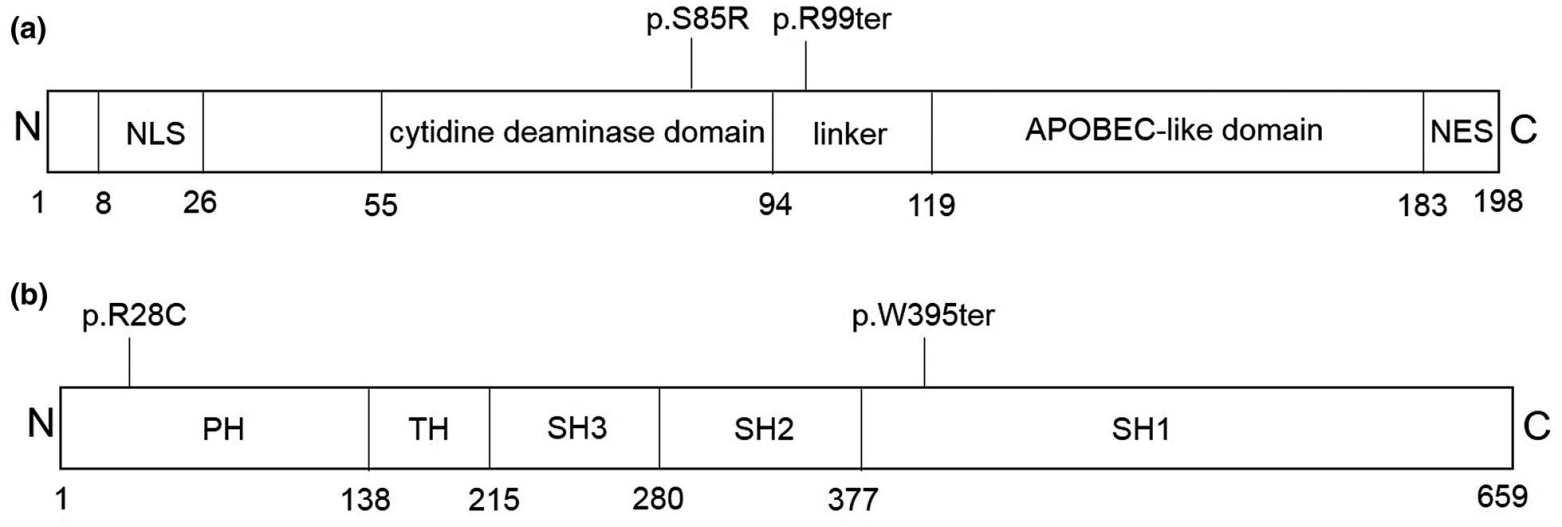
Linear map of the mutations in AICDA (a) and BTK (b). NES, nuclear export signal; NLS, nuclear localization signal; PH, pleckstrin homology domain; SH1, catalytic kinase domain; SH2, Src homology 2 domain; SH3, Src homology 3 domain; TH, Tec homology domain

**FIGURE 3:**
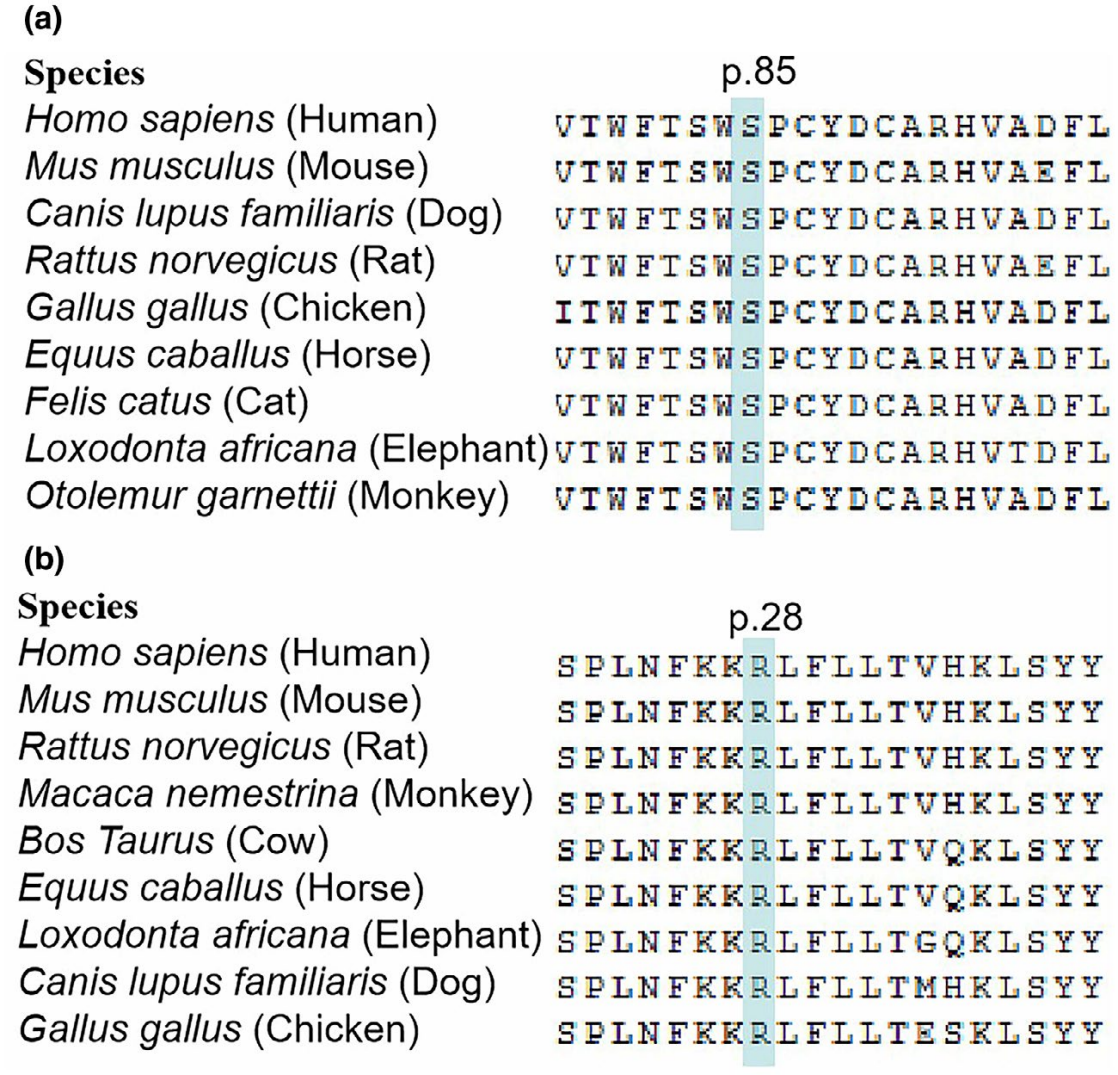
Conservation analysis of the two substituted amino acids in AICDA (a) and BTK (b)

**FIGURE 4:**
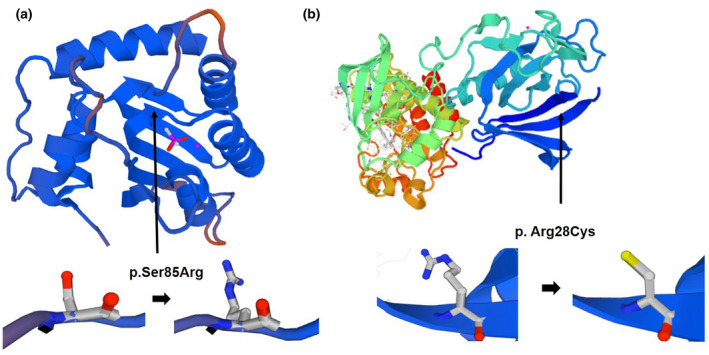
Simulation of the conformation changes caused by amino acid substitutions in AICDA (a) and BTK (b)

The c.255C>A variant results in the replacement of a hydrophilic serine with an alkaline arginine at position 85 in the cytidine deaminase domain of the AICDA protein, while the c.295C>T variant causes the peptide to terminate in the linker region (Figure 2a). The c.1185G>A variant in *BTK* leads to early termination of translation, with the resulting protein lacking the entire catalytic kinase domain, while the c.82C>T alteration results in the substitution of an alkaline arginine residue with a hydrophilic cysteine at position 28 in the PH domain (Figure 2b). This amino acid replacement has been reported previously in several patients with XLA in China and other countries (Vihinen et al., 1997; Zhang et al., 2014). Both of the Ser85 residue in AICDA and Arg28 in XLA are highly evolutionarily conserved amino acids (Figure 3a,b). Further, both of these missense variants are predicted to be deleterious using the SIFT and PolyPhen‐2 prediction tools, and were assessed to likely decrease protein stability using I‐Mutant2.0. Moreover, significant amino acid and polypeptide conformation changes were observed on the generation of a simulation using SWISS‐MODEL (Figure 4a,b).

### 3.3 Evaluation of molecular pathology according to the American College of Medical Genetics and Genomics (ACMG) guidance

According to the standards and guidelines for the interpretation of sequence variants developed by the ACMG (Richards et al., 2015). The c.255C>A (p.Ser85Arg) variant is likely pathogenic, with one strong (PS1) and four supporting (PP1, PP2, PP3, PP4) pieces of evidence for pathogenicity. The c.295C>T (p.Arg99ter) alteration is classified as a pathogenic variant, with one very strong (PVS1), one moderate (PM2), and three supporting (PP1, PP3, PP4) pieces of evidence for pathogenicity. Further, the c.82C>T (p.Arg28Cys) variant was evaluated as a pathogenic variant due to two strong (PS1, PS3; Bajpai et al., 2000) pieces of evidence for pathogenicity. The c.1185G>A (p.Trp395ter) variant was considered pathogenic due to one very strong (PVS1) and one strong (PS1; Gofshteyn et al., 2016) piece of evidence for pathogenicity.

## 4 DISCUSSION

Hyper‐IgM syndrome (HIGM) is a group of immunodeficiency disorders associated with elevated levels of IgM. Type 2 (HIGM2) caused by the deficiency of *AICDA*, is a very rare autosomal recessive disease (de la Morena, 2016). *AICDA* maps to chromosome 12p13 and encodes an activation‐induced cytidine deaminase, which is an RNA‐editing enzyme specifically expressed in activated B cells in the centers of secondary lymphoid organs (Muto et al., 2000). This expression initiates class switch recombination and somatic hypermutation by inducing DNA lesions in targeted DNA sequences in the S and V regions of immunoglobulin genes (Durandy et al., 2006). The AICDA protein contains a cytidine deaminase domain and an apolipoprotein B editing the complex (APOBEC)‐like domain, connected by a linker domain. Additionally, a nuclear localization signal (NLS) and nuclear export signal (NES) are located at the C‐ and N‐termini of the protein, respectively. Mutations that cause HIGM2 have been found in all domains of the AICDA protein. The c.255C>A mutation reported in this study led to the replacement of hydrophilic serine by alkaline arginine at position 85, and another transversion to hydrophilic asparagine at the same site caused by 254G>A has been previously found in HIGM patients (Durandy et al., 2006). The c.295C>T variant leads to the early termination of the peptide in the linker region. Several other nonsense mutations that cause terminations at latter regions than c.295C>T have been reported to cause disease (Mahdaviani et al., 2012). According to the above analysis, both of the two novel variations in AICDA disrupt important functional domains of the protein, thereby causing HIGM2 in their compound heterozygous state.

XLA is characterized by the absence of all immunoglobulin isotypes and B cells, and is caused by mutations in *BTK*, which maps to chromosome Xq21.3‐22. The encoded BTK protein is expressed in all cells originating from hematopoietic stem cells, except T lymphocytes and plasma cells. BTK acts in the signal transduction pathway downstream of the B cell antigen receptor and has an important role in B cell development and antigen‐receptor signaling (Hendriks et al., 2014; Melchers et al., 2000; Pal et al., 2018). The BTK protein contains five distinct structural domains including pleckstrin homology (PH), Tec homology (TH), Src homology 3 (SH3), Src homology 2 (SH2), and catalytic kinase (SH1) domains (Aadam et al., 2016). Among the large number of the XLA‐causing variants in BTK, Arg28 in the PH domain is a hotspot (Chen et al., 2016); two variants (Arg28His and Arg28Cys) have been reported at this site, where Arg28Cys was found to cause less severe impairment and protein dysfunction than other amino substitutions (Kojima et al., 1997). The Trp395ter (rs1064796836) nonsense variant in the BTK protein has previously been reported as associated with XLA (Gofshteyn et al., 2016). This variant is predicted to cause loss of normal protein function, either through protein truncation or nonsense‐mediated mRNA decay. Moreover, a previous study found that BTK expression was reduced in patients with stop codon mutations, but not in those with missense mutations (Teocchi et al., 2015). In our study, patient no. 2, who carried the c.82C>T (p.Arg28Cys) variant, had milder clinical features than patient no. 3, with the c.1185G>A (p.Trp395ter) alteration, consistent with previous reports.

As they are both immunodeficiency diseases, HIGM2 and XLA can share common clinical phenotypes, such as recurrent and persistent sinopulmonary infection. Diagnosis of the two diseases can be conducted based on patient immunophenotype. Patients with HIGM2 invariably have typically high IgM, and low IgG and IgA function as a result of abnormal switch recombination, while some patients may generate normal IgM in early childhood. Patients with XLA generally have very low circulating B cell numbers and antibodies are almost completely absent in the serum, due to a severe block of B cell development in the bone marrow (Anon. 1997; Pal et al., 2018). XLA should be considered in male patients with such features. Despite these differences between HIGM2 and XLA, the presence of ambiguous or atypical phenotypes in some cases makes it difficult to provide a definite diagnosis based on biochemical indices alone. Given the strong relationship between infection in early life and mortality from PIDDs, genetic tests are necessary to make faster and more accurate diagnoses for patients with these conditions (Heimall et al., 2018).

Immunoglobulin replacement therapy is the standard treatment for patients with antibody deficiencies due to PIDDs (Hendriks et al., 2011; Krivan et al., 2017). Other treatments, using transfer factors, antibiotics, antifungal drugs, and antiviral drugs, can also prevent infections; however, poor response to antibiotic treatment or drug side effects may lead to life‐threatening complications and compromise patient quality of life. Given these challenges, the development of stem cell transplantation technology and gene therapy has the potential to offer a life‐long effective therapy, reflected in better prognoses for patients with PIDDs (Fox et al., 2018; Marciano & Holland, 2017). This study has two potential limitations. First, as the missense mutation in *AICDA* is evaluated as a likely pathogenic variant, in vitro or in vivo functional studies should be established to support its pathogenicity. Second, detailed lung high‐resolution computed tomography features and specific antibody responses are not discussed.

## 5 SUMMARY

Pathogenic variants in *AICDA* and *BTK*, two of which were novel and one known, were confirmed in three Chinese patients with PIDDs using a combination of genetic and biochemical tests. These results enrich the mutation spectrum of PIDDs worldwide and will help to facilitate the diagnoses of patients with PIDDs at an early age.

## CONFLICT OF INTEREST

None declared.

## Supporting information

Table S1

## Data Availability

The data that support the findings of this study are openly available in figshare at http://doi.org/10.6084/m9.figshare.13174292, reference number (MGG3‐2020–05–0703.R2).
