# Supplementary material for: Novel mutations in hyper‐IgM syndrome type 2 and X‐linked agammaglobulinemia detected in three patients with primary immunodeficiency disease
Source: Mol Genet Genomic Med. 2020 Dec 30;9(1):e1552. doi: 10.1002/mgg3.1552 (PMC7963428; doi:10.1002/mgg3.1552)
Supplement: Supplementary file 1 — Table S1 [file MGG3-9-e1552-s001.docx]

**Table S1** Genes related to primary immunodeficiency diseases with antibody defects

| **Hypotypes** | **Genes** | | | |
| --- | --- | --- | --- | --- |
| Absence or significantly reduction of all immunoglobulin isotypes and peripheral B lymphocytes | *BTK* | *IGLL1* | *CD79A* | *CD79B* |
|  | *BLNK* | *PIK3R1* | *TCF3* |  |
| Significantly reduction of over two kinds of immunoglobulin isotypes, with normal or reduced peripheral B lymphocytes | *ICOS* | *CD19* | *CD81* | *MS4A1* |
|  | *CR2* | *TNFRSF13B* | *LRBA* | *TNFRSF13C* |
|  | *TNFSF12* | *NFKB2* | *CXCR4* |  |
| Significantly reduction of serum IgG and IgA, but normal or increased IgM, with normal peripheral B lymphocytes | *CD40LG* | *CD40* | *AICDA* | *UNG* |
| Abnormal immunoglobulin levels with reduced peripheral B lymphocytes | *PRKCD* | | *PIK3CD* | |
| Deficiency of specific antibodies with normal immunoglobulins and peripheral B lymphocytes | *IL7R* | | | |
| Transient hypogammaglobulinemia of infancy with normal peripheral B lymphocytes | *CD19* | | | |
